# Effect of growing temperature on root carbohydrate content and postharvest asparagus tip breakdown

**DOI:** 10.1002/jsfa.14172

**Published:** 2025-02-06

**Authors:** Emma R. Collings, M. Carmen Alamar, Leon Terry

**Affiliations:** ^1^ Postharvest Research Group, Cranfield University Bedfordshire UK; ^2^ Cobrey Farms Ross‐on‐Wye Herefordshire UK

**Keywords:** *Asparagus officinalis*, asparagine, sugars, storage

## Abstract

**BACKGROUND:**

Tip breakdown has been identified as the main issue causing deterioration in asparagus quality during storage; however, the underlying mechanisms responsible for its development are unknown. Previous work showed higher incidence of tip breakdown occurring later in the season, when growing temperature was higher. Spears from two growing conditions (cooler vs warmer) were harvested throughout the season to assess tip breakdown incidence and quality attributes (asparagine and non‐structural carbohydrates) during storage, with the aim of enhancing understanding and identifying potential biomarkers of this physiological disorder. Root samples were also collected just after harvest to determine if storage root carbohydrate content was associated with a predisposition to tip breakdown.

**RESULTS:**

Rapid growth due to warmer temperatures (up to 45 °C) resulted in spears with lower sugar content and higher incidence of tip breakdown in comparison with cooler conditions. Asparagine slowly increased through the season (7 to 11 mg g^−1^ DW) with no differences between growing conditions, suggesting that it is not a biomarker for tip breakdown. Pre‐season spears (warm temperature only) had double the amount of sugar in comparison with early season spears, with no incidence of tip breakdown despite an extended storage period (up to 18 days at 7 °C). Sugar concentration in roots was similar between growing conditions and between pre‐ and early season despite clear differences in spear sugar content.

**CONCLUSION:**

These results showed a strong positive link between cooler growing conditions, high spear sugar content, and low susceptibility to tip breakdown, which was not reflected in root sugar concentrations. However, further research is needed to understand the molecular mechanisms responsible for tip breakdown. © 2025 The Author(s). *Journal of the Science of Food and Agriculture* published by John Wiley & Sons Ltd on behalf of Society of Chemical Industry.

## INTRODUCTION

Asparagus is a high‐value crop with a limited shelf life. Significant food loss occurs during periods of rapid growth due to the absence of suitable storage methods.^1^ Studies have identified tip breakdown as a limiting factor during storage^2^, ^3^ and, more recently, as the main limiting factor preventing commercialization of controlled‐atmosphere (CA) storage.^1^ Tip breakdown, also known erroneously as tip rot, is characterized by the presence of soft darkening tips (Supporting Information, Fig. S1), followed by water‐soaked bracts (Supporting Information, Fig. S1) and a foul odor towards the latter stages.^4^ Most of the work investigating the causes of tip breakdown was performed over 10 years ago (Table 1).^1^, ^2^, ^3^, ^4^, ^5^, ^6^, ^7^, ^8^, ^9^, ^10^, ^11^ Since then, technology and pre‐ and post‐harvest methods have advanced, which could now enable researchers to gain a mechanistic understanding of the development of tip breakdown.

**Table 1:** Overview of studies reporting tip breakdown in asparagus

Reference	Factors tested	Outcomes
5	Fumigation	Enhanced symptoms of tip breakdown and other senescent symptoms including butt rot, wilting, and stem lesions.
2	Controlled atmosphere (CA)	Controlled atmosphere had no effect on the incidence of tip breakdown
6	Carbohydrate reserves	Lower incidence of tip breakdown observed in spears with higher levels of carbohydrate.
7	2 Bacteria invasion 3 Soil moisture 4 Variability between plants 5 Seasonal factors 6 Storage temperatures and time	Tip breakdown is a physiological disorder Bacteria are secondary invaders High prevalence plants had lower soluble carbohydrate levels Unknown why cultivars have different susceptibility Tip breakdown incidence increases as harvest season progresses
8	Protein synthesis inhibitors (actinomycin D or cycloheximide)	Inhibitors accelerated postharvest senescence Spear elongation reduced Tips had lower concentrations of hexose and asparagine No increase in ammonia Increase in tip breakdown incidence Exhaustion of tip soluble carbohydrates and abnormal (reduced) asparagine metabolism linked to tip breakdown
4	Temperature during growth	Spears grown at 20 °C had higher frequency of tip breakdown (99%) Spears grown at 13 °C had lower frequency of tip breakdown (3%) Tip breakdown negatively correlated with sucrose content of spear tip Tip breakdown positively correlated with tip respiration and spear height growth rate Sucrose in tip at harvest best indicator of predisposition to tip breakdown but not necessarily the primary cause.
9	Genetic variation in tip breakdown	Ultrastructural studies showed the disorder has a physiological origin commencing with floral buds and bracts in the spear tip Bacteria did not invade until 4–5 days after first signs of breakdown were evident. Genotypic differences in tip breakdown expression observed.
10	Physical damage Washing Cooling treatments Delays in cooling after harvest	Method of cooling and delays after harvest (up to 12 h) did not affect tip breakdown incidence. Tip breakdown increased with increasing severity of non‐visible impact damage to the tip. Tip breakdown incidence exacerbated by washing (after damage) and exploited further by microorganisms (*Fusarium* sp.).
11	Early (cooler) vs late (warmer) season Early morning harvest vs afternoon harvest Short vs tall Carbohydrate reserves	Early season (cooler) were less susceptible to tip breakdown Time of day had no effect Short spears less susceptible Tall spears had lower levels of soluble carbohydrate and more axillary buds than short spears. Describes first signs of tissue damage in floral buds Microbial growth developed on the surface of damaged tissue.
3	Edible coating	Coatings did not delay incidence of tip breakdown, but chitosan reduced foul odor suggesting anti‐microbial activity of chitosan.
1	Controlled atmosphere (CA)	All three CA conditions retained quality parameters (texture, color, moisture content, and visual appearance) but it did not suppress tip breakdown.

This table summarizes the research work carried out during the last 30 years on postharvest asparagus tip breakdown, including the factors tested and the main outcomes of the investigation.

Previous research reported that tip breakdown is a physiological disorder. Bacteria species present on spears affected by tip breakdown may include *Erwinia*, *Pseudomonas* and general soil bacteria. As a single causative organism could not be isolated, Koch's first postulate was not satisfied and, thus, bacteria were considered secondary invaders.^7^ Furthermore, pre‐ and post‐harvest methods to prevent microbial growth, such as fungicides and agricultural antibiotics, did not reduce postharvest tip breakdown incidence.^5^, ^7^ This suggests that other factors are involved, which may alter the physiology of the spears, predisposing them to tip breakdown.

Jermyn *et al*.^9^ first reported the floral buds of the tip to be the initial site for tip breakdown to appear. During warmer growing conditions, typically towards the mid and late season, these floral buds under the bracts are likely to grow at an increased rate due to the plants’ greater propensity to fern. A higher susceptibility to tip breakdown has been linked to fast growth rate (tall spears) during warmer temperatures^4^, ^11^ as well as low carbohydrate content in the tip.^6^, ^8^, ^12^ It is therefore possible that depletion of carbohydrate levels in the asparagus crown as the harvest season progresses may contribute to increased spear susceptibility to tip breakdown. However, as far as the authors of the current study are aware, there has been no literature investigating the relation between root carbohydrate reserves and spear sugar content on the development of tip breakdown.

Asparagus spears grow from bud clusters found on fleshy storage roots attached to the rhizome (crown). These roots continue to grow throughout the lifespan of the asparagus plant (15 years and more), whereas smaller feeder roots (attached to storage roots) are short lived.^13^, ^14^ Spear diameter attributed to primary and secondary spear emergence from a bud cluster^13^ could affect predisposition to tip breakdown as primary spears, which have larger spear diameters, dominate the early harvest season when incidence of tip breakdown is low (personal communication, Cobrey Farms). The carbohydrate reserves in the storage roots/crowns and the ability to mobilize them to actively metabolize tissue in the tip, particularly in secondary spears, which are already likely to have lower carbohydrate content compared to primary spears, could be another possible contributing factor linked to tip breakdown. Overall, and despite previous research on tip breakdown, the true underlying mechanism responsible for tip breakdown is still unknown.

This work hypothesizes that, as the season progresses, warmer weather increases spear growth, causing more rapid consumption of nutrients, the content of which is already likely to be lower in secondary spears than in primary spears. This, in turn, will result in a higher incidence of postharvest spear tip breakdown.

To test these hypotheses, the relationship between (i) the spear diameter (small vs large) and spear carbohydrate content, (ii) the changes of spear and root carbohydrate content throughout the harvest season, and (iii) the impact of spears grown under warmer and cooler temperatures were investigated and compared with the development of postharvest tip breakdown.

## MATERIALS AND METHODS

### Plant and root material

Spears (cv. Gijnlim) were harvested in the UK by hand between 7:30 a.m. and 9:00 a.m., at three time points – early (end of April), mid (end of May) and late season (end of June) – throughout the 2019 season, from two growing conditions (cooler versus warmer), which consisted of spears grown in open‐field conditions (termed ‘cooler’) while the other spears had been grown under a polytunnel (termed ‘warmer’) (Table 2). Spears grown under warmer conditions were sampled 2 weeks prior to the harvest spears grown under cooler conditions, due to the earlier emergence of spears (referred to as pre‐season harvest). To assess differences between grades, spears were sorted by hand into two sizes consisting of small (ca. 7 to 11 mm diameter) and large (ca. 14 to 18 mm diameter), measured at 15 cm from the tip.

**Table 2:** Sampling time points during storage for roots and spears collected from cooler (open‐field) versus warmer (polytunnel) growing conditions during the harvest season 2019

Season	Date harvested (2019)	Sample collected	Growing condition	Storage time (days)	Total storage duration (days)
Pre	5 April	Spears	Warmer only	7 CS + 11 SL	18
	10 April	Roots	Cooler versus warmer	N/A	N/A
Early	22 April	Spears	Cooler versus warmer	7 CS + 11 SL	18
	24 April	Roots	Cooler versus warmer	N/A	N/A
Mid	21 May 2019	Spears	Cooler versus warmer	7 CS + 9 SL	16
	20 May 2019	Roots	Cooler versus warmer	N/A	N/A
Late	17 June 2019	Spears	Cooler versus warmer	7 CS + 8 SL	15
	18 June 2019	Roots	Cooler versus warmer	N/A	N/A

Spears were subsequent cold stored (CS) at 1 °C followed by storage at shelf‐life (SL) conditions (7 °C).

Immediately after harvest, spears were transferred to refrigerated conditions within 2–3 h. Spears were kept overnight at 1 °C before transportation to Cranfield University within 3 h by refrigerated transport (at 4 °C). Between 2 to 3 days after spears were harvested, root samples were collected at 15 cm from the crown zero line from 0.15–0.3 m depth using a bi‐partite root corer. In total, 160 root samples were collected at each time point. Root samples were stored under refrigerated conditions (4 °C) within 2–3 h after harvest, before being packaged in a polystyrene box with ice packs (care was taken to ensure the ice packs did not have direct contact with the root samples) for delivery to the Postharvest Research Group, Cranfield University. Upon arrival at the laboratory (within 5–6 h), roots were washed, snap frozen in liquid nitrogen, and freeze dried. Samples were then powdered using a homogenizer (Precellys 24, Stretton Scientific Ltd, Derbyshire, UK) at 5000 rpm for 20 s using ceramic beads. The freeze‐dried powder was stored at −40 °C prior to biochemical analysis.

### Physiological analysis

Sampling occurred regularly throughout shelf life, and the exact time of each time point was determined during the experiment by evaluating the quality of the spears visually. After visual assessment at each time point, spear tips (0–4 cm from top) were snap frozen and stored at −80 °C for subsequent biochemical analysis.

#### Subjective assessment

Spears were also inspected visually for the presence of tip breakdown on removal from cold storage and after the end of shelf‐life, and results were expressed in percentage of spears per treatment, according to Collings *et al*.^15^ Briefly, the top 4 cm of the spear tip were cut in half longitudinally to assess internally, and classified using a scale from 0 to 5, where 0 had no tip breakdown and 5 had severe tip disintegration.

### Biochemical analysis (non‐structural carbohydrates and l‐asparagine)

Spears (after physiological analysis) and roots were snap frozen immediately in liquid nitrogen and stored at −80 °C, before subsequent freeze drying and powdering in a homogenizer (Precellys 24, Stretton Scientific Ltd) at 5000 rpm for 20 s using ceramic beads. The freeze‐dried powder was stored at −40 °C. Non‐structural carbohydrates and the amino acid l‐asparagine were extracted according to the method described in Anastasiadi *et al*.^16^ Briefly, freeze‐dried samples were extracted in 62.5% (v/v) aqueous methanol solvent in a shaking water bath at 55 °C for 15 min. Extracts were cooled, filtered through a 0.2 μm nylon filter and diluted (1:1 v/v) with high‐performance liquid chromatography (HPLC) water before analysis on a HPLC fitted with an evaporative light‐scattering detector (ELSD).

### Statistical analysis

Statistical analyses were carried out using Statistica for Windows version 10, 64‐bit (StatSoft Inc., Tulsa, OK, USA). A general linear model multi‐way analysis of variance (ANOVA) was used to identify significant differences (*P* < 0.05) followed by Fisher's *post hoc* test to assess least significant differences (LSD) between main factors and their interactions. Standard errors (SE) for each mean are displayed in the figures and tables and represent the standard error estimated from the residual mean square.

## RESULTS AND DISCUSSION

### Season progression and warm temperatures increased incidence of tip breakdown

**Figure 1:**
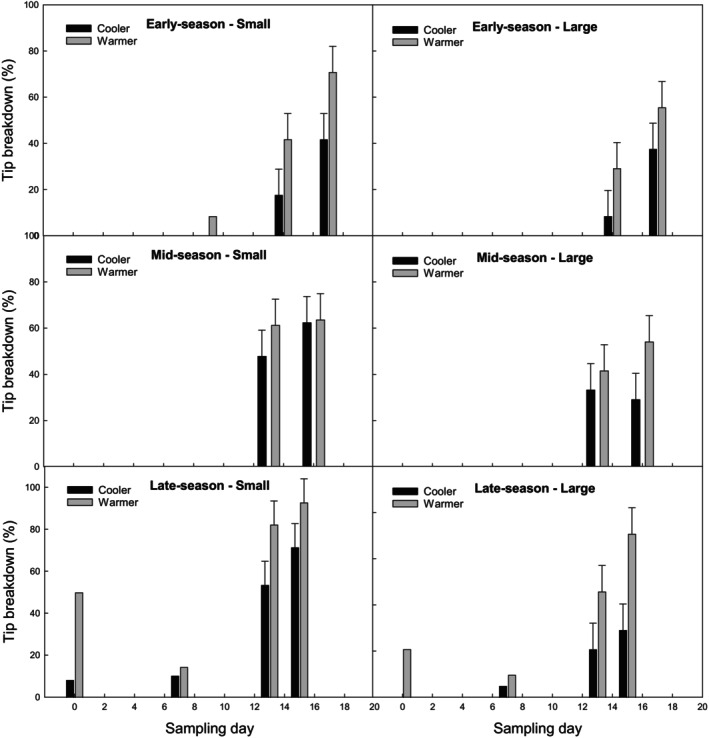
Incidence of postharvest asparagus tip breakdown (cv. Gijnlim). Tip breakdown incidence (%) in small (7–11 mm diameter) and large (14–18 mm diameter) grade spears grown under cooler (open‐field) and warmer (polytunnel) conditions. Incidence was recorded during storage (7 days at 1 °C, followed by 0–18 days of simulated shelf‐life at 7 °C) for three harvests: early (late April), mid (late May), and late season (late June). Statistical analysis was conducted only for the last two storage days per season, as no breakdown occurred earlier. Standard error of the mean (± SE) is shown for these sampling points. No significant interaction was found between storage time, growing condition, season, and grade (*P* > 0.05). However, least significant differences (LSDs) were significant (*P* < 0.05) for season (11.2), storage time (9.1), growing condition (9.1), and grade (9.1). Total number of observations: 96.

**Figure 2:**
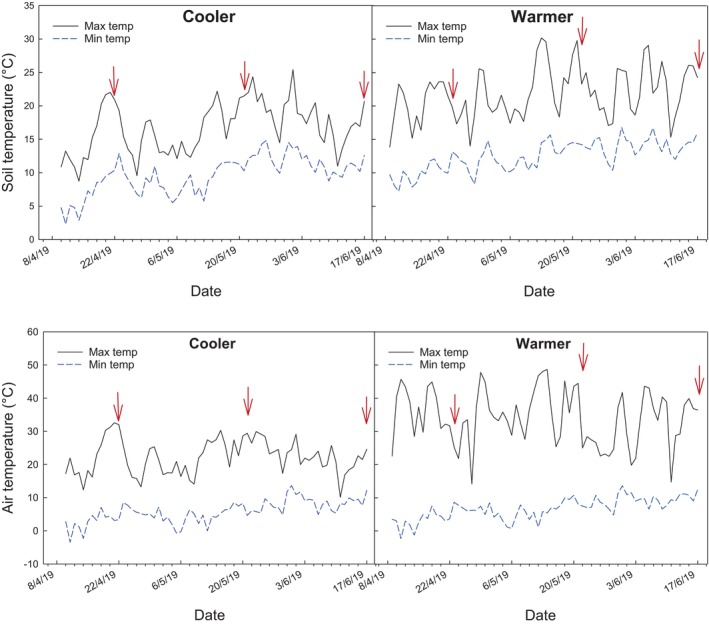
Soil and air temperature during asparagus growing season. Daily maximum (shown as solid black line) and minimum (dashed blue line) soil (A) and air (B) temperature during the asparagus harvest season for cooler (open‐field) and warmer (polytunnel) growing conditions. Red arrows indicate when early, mid, and late‐harvest samples were harvested for postharvest laboratory trials.

Assessment of external tip breakdown revealed that an increasing percentage of spears was affected as the harvest season progressed (Fig. 1). Soil and air data loggers showed there was a clear distinction in temperature between the different growing conditions (cooler vs warmer) (Fig. 2). Interestingly, in pre‐season spears (both grades) grown under warm conditions, no incidences of tip breakdown were observed. As far as the authors are aware, research on pre‐season asparagus in relation to tip breakdown is scant.

After 13 or more days of storage, the overall incidence of tip breakdown (irrespective of grade) was higher in late season (58%), followed by mid‐season (49%) and early season (36%) (Table 3; LSD_Season_: = 11.2), as well as under warmer conditions (58%) in comparison with cooler conditions (38%) (Table 3; LSD_growing condition_ = 9.1). For early season harvested asparagus, spear tip breakdown was only observed towards the end of shelf life. Irrespective of grade, tip breakdown in early season was significantly higher in spears grown under warmer conditions than those grown under cooler conditions (13% vs 35%, and 64% vs 40%, after 15 and 17 days shelf‐life, respectively) (Fig. 1). These early season spears were harvested when air temperatures for cooler and warmer conditions reached between ca. 31–28 °C (soil temperatures ca. 23–21 °C) (Fig. 2). However, for mid‐ and late‐season harvests, spears were collected when temperatures inside the polytunnel (warmer conditions) had reached the maximum air temperature of 45 and 40 °C (soil temperatures 28 and 25 °C) in comparison with 30 and 23 °C for open‐field (cooler conditions; soil temperature 22 and 21 °C), respectively. Despite this, growing conditions did not influence tip breakdown development in mid‐season spears and values increased progressively during storage reaching up to 60%. Based on our results, it could be possible that, predisposition and incidence of tip breakdown depends on air temperatures. However, additional work would be required to confirm what woudl be the treshhold, if any, that may trigger tip breakdown predisposition.

**Table 3:** Non‐structural carbohydrates and asparagine concentration in ‘Gijnlim’ asparagus tips grown under warmer growing conditions.

Season	Storage time (days)	Grade	Fructose (mg g^−1^)	Fructose ± SD	Glucose (mg g^−1^)	Glucose ± SD	Sucrose (mg g^−1^)	Sucrose ± SD	Asparagine (mg g^−1^)	Asparagine ± SD
Pre	0	Small	57.01	*2.18*	39.84	*1.37*	84.60	*5.42*	6.53	*2.05*
Pre	0	Large	61.67	*2.18*	39.76	*1.37*	93.75	*5.42*	6.31	*2.05*
Pre	7	Small	33.23	*2.18*	19.09	*1.37*	56.61	*5.42*	6.36	*2.05*
Pre	7	Large	42.04	*2.18*	22.27	*1.37*	73.22	*5.42*	6.94	*2.05*
Pre	18	Small	21.99	*2.18*	23.94	*1.37*	160.08	*5.42*	57.47	*2.05*
Pre	18	Large	23.96	*2.18*	22.22	*1.37*	131.13	*5.42*	46.09	*2.05*
Early	0	Small	23.76	*2.18*	15.90	*1.37*	33.37	*5.42*	8.39	*2.05*
Early	0	Large	24.57	*2.18*	15.89	*1.37*	34.81	*5.42*	8.84	*2.05*
Early	7	Small	9.73	*2.18*	8.06	*1.37*	22.78	*5.42*	8.55	*2.05*
Early	7	Large	9.60	*2.18*	6.86	*1.37*	28.39	*5.42*	9.92	*2.05*
Early	17	Small	7.28	*2.18*	9.26	*1.37*	53.57	*5.42*	37.67	*2.05*
Early	17	Large	5.65	*2.18*	7.63	*1.37*	57.94	*5.42*	43.45	*2.05*
Mid	0	Small	22.62	*2.18*	9.84	*1.37*	29.98	*5.42*	9.40	*2.05*
Mid	0	Large	20.69	*2.18*	8.91	*1.37*	33.15	*5.42*	11.02	*2.05*
Mid	7	Small	4.49	*2.18*	4.04	*1.37*	6.19	*5.42*	9.84	*2.05*
Mid	7	Large	5.83	*2.18*	3.26	*1.37*	8.93	*5.42*	15.83	*2.05*
Mid	16	Small	3.08	*2.18*	5.59	*1.37*	40.14	*5.42*	38.47	*2.05*
Mid	16	Large	2.03	*2.18*	4.09	*1.37*	28.90	*5.42*	41.60	*2.05*
Late	0	Small	19.44	*2.18*	8.94	*1.37*	7.93	*5.42*	5.49	*2.05*
Late	0	Large	32.26	*2.18*	13.90	*1.37*	13.02	*5.42*	15.15	*2.05*
Late	7	Small	6.07	*2.18*	5.96	*1.37*	11.44	*5.42*	11.77	*2.05*
Late	7	Large	7.47	*2.18*	5.35	*1.37*	13.44	*5.42*	14.25	*2.05*
Late	15	Small	3.10	*2.18*	6.81	*1.37*	24.66	*5.42*	33.22	*2.05*
Late	15	Large	3.35	*2.18*	5.04	*1.37*	30.91	*5.42*	36.32	*2.05*
LSD: Season			***2.8	‐	***1.8	‐	***7.0	‐	ns (*P* > 0.05)	‐
LSD: storage time (days)			***2.4	‐	***1.5	‐	***6.1	‐	***2.3	‐
LSD: Grade			*2.0	‐	ns (*P* > 0.05)	‐	ns (*P* > 0.05)	‐	*1.9	‐
LSD: Season × storage time (days)			***4.9	‐	***3.1	‐	***12.1	‐	***4.6	‐
LSD: Season × storage time (days) × grade			ns (*P* > 0.05)	‐	ns (*P* > 0.05)	‐	ns (*P* > 0.05)	‐	ns (*P* > 0.05)	‐

Effect of storage time on sugar and asparagine content in asparagus tips. Impact of storage time (7 days at 1 °C, followed by shelf‐life at 7 °C) on fructose, glucose, sucrose, and asparagine content (mg g⁻¹ DW) in asparagus tips from pre‐season (early April), early (late April), mid (late May), and late season (late June) harvests grown under warmer conditions. Standard error of the mean (± SE) is shown in italics for each data point. Least significant difference (LSD) values are provided for each significant main factor and interaction (*P* < 0.05). Significance levels: ns = not significant; *P < 0.05; **P < 0.01; ***P < 0.001. Total number of observations: 96.

Tip breakdown in late‐season spears increased during storage but values were again significantly higher overall in spears grown under warmer conditions (at 58%) compared with cooler conditions (at 38%) (Fig. 1). Moreover, small spears were approximately twofold more susceptible to develop tip breakdown than large spears. It was also found that spears harvested in the late season had tip breakdown at harvest (day 0) and after 1 week of cold storage (day 7) (data not included in statistical analysis), which was not observed for early and mid‐season crops. These results agree with findings reported by others^4^, ^11^ and support the hypothesis that warmer temperatures/faster growth increase the incidence of tip breakdown. However, due to a limited number of spears available and with the increase in tip breakdown in spears grown under warmer conditions during late season, some spears were likely to have had tip breakdown at the time they were placed into storage. This would explain the observed higher percentage of tip breakdown noted at day 0 whereas a lower percentage was recoded on day 7. This decrease is more likely due to sample variation as opposed to a decrease in tip breakdown during storage.

### Spear emergence unlikely to be a factor contributing towards predisposition to tip breakdown

Incidence of tip breakdown was found to be significantly higher overall in small‐grade spears (48%) than large‐grade ones (25%). Plant age has been found to have a slight effect on spear weight and diameter;^17^ however, the asparagus crowns used for this trial were all similar in age (2 years old) and were planted at the same time. As far as the authors of this study are aware, this is the first time the effect of grade on tip breakdown has been reported. Lill and Borst^11^ did report differences in tip breakdown between different‐sized spears where significantly higher percentages were observed in tall spears than short ones. That said, it is difficult to compare both pieces of research directly because, in the current study, spear length was not recorded at harvest.

In the UK, primary spears (from primary buds on a bud cluster), which form large buds on the base of bud clusters,^18^ dominate the early season crop when tip breakdown is lowest (personal communication, Cobrey Farms). These spears tend to be slower growing with larger spear girth. In contrast, secondary spears, emerge around the middle of the season (coinciding with the warmer weather) and generally have smaller diameters. Spear diameter has been linked to crown age;^17^ however, as all spears were harvested from crowns of a similar age this is unlikely to have had an effect. The likely presence of a higher number of secondary spears in mid and late harvests could explain the greater incidence of tip breakdown towards the end of the season and the differences between grades. However, spear diameter size was not found to influence sugar profiles because small (ca. 7 to 11 mm diameter) and large (ca. 14 to 18 mm diameter) spears had similar fructose (14–15 mg g^−1^), glucose (10–11 mg g^−1^), and sucrose (31–34 mg g^−1^) content despite having higher and lower tip breakdown, respectively (data not shown). These results suggest primary and secondary spear emergence from a bud cluster may not influence the predisposition to tip breakdown. That said, it was difficult to monitor the emergence of primary or secondary spears accurately without observing growth directly from the crown. Furthermore, other factors, including low soil moisture content, can influence spear diameter;^19^ classification based on grade alone may not, therefore, have identified primary and secondary spears correctly. To overcome this, more detailed harvest studies would be required to confirm whether tip breakdown is associated with secondary spears.

### Lower sugar concentration is related to higher incidence of postharvest spear tip breakdown

**Figure 3:**
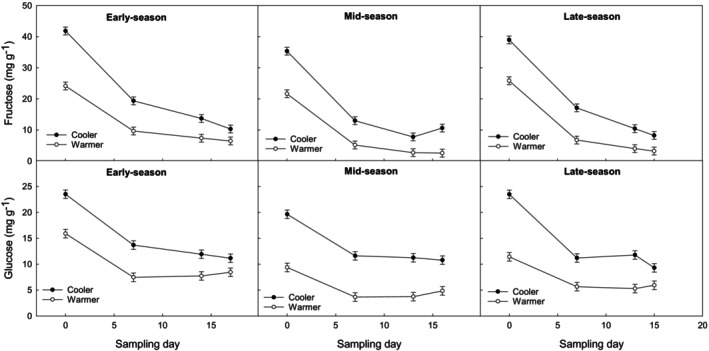
Fructose and glucose concentration in ‘Gijnlim’ asparagus tips. Effect of storage time (7 days at 1 °C, followed by 0–18 days of simulated shelf‐life at 7 °C) on fructose and glucose content (mg g⁻¹ DW) in asparagus tips from early (late April), mid (late May), and late‐season (late June) harvests, grown under cooler and warmer conditions. Spear grades were combined due to a lack of significant differences. Standard error of the mean (± SE) is shown for each data point. No significant interaction was found between storage time, growing condition, and season (*P* > 0.05). Least significant differences for significant main factors and interactions (*P* < 0.05) were as follows: fructose – growing condition (1.2), season (1.5), storage time (1.7), and storage time × growing condition (2.5); glucose – growing condition (0.8), season (1.0), storage time (1.1), and storage time × growing condition (1.6). Total number of observations: 192.

**Figure 4:**
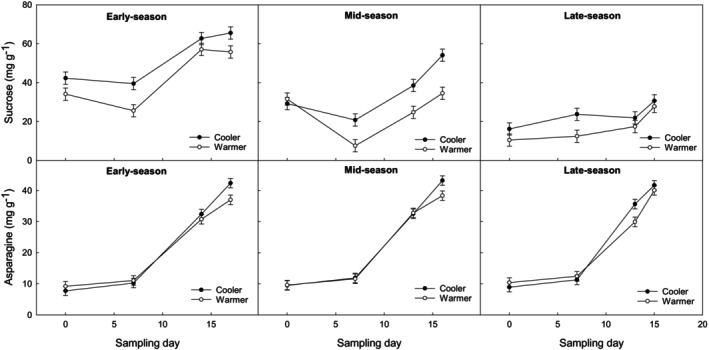
Sucrose and asparagine concentration in ‘Gijnlim’ asparagus tips. Effect of storage time (7 days at 1 °C, followed by 0–18 days of simulated shelf‐life at 7 °C) on sucrose and asparagine content (mg g^−1^ DW) in asparagus tips from early (late April), mid (late May), and late‐season (late June) harvests, grown under cooler and warmer conditions. Spear grades were combined due to a lack of significant differences. Standard error of the mean (± SE) is shown for each data point. No significant interaction was found between storage time, growing condition, and season (*P* > 0.05). Least significant differences for significant main factors (*P* < 0.05) were as follows: sucrose – growing condition (3.1), season (3.8), and storage time (4.4); asparagine – growing condition (1.4), season (1.8), and storage time (2.1). Total number of observations: 192.

The monosaccharides glucose and fructose decreased throughout postharvest storage by twofold and fourfold for warmer and cooler growing conditions, respectively, regardless of harvest season (Figs 3 and 4 and Table 3). This agrees with Anastasiadi *et al*.^1^, ^12^, ^16^ Moreover, the steady decline in glucose and fructose during storage coincided with the increase in tip breakdown incidence (Table 1). It should be noted that tip breakdown samples and healthy samples were combined within a replicate during sampling, which may have introduced some bias to the sugar results. For future studies, healthy and tip breakdown spears will be separated prior to biochemical analysis.

Non‐structural carbohydrate analysis of spear tips showed clear differences in sugar profiles between warmer conditions and cooler ones. As an overall mean value across the whole harvest period (early, mid, and late‐season), fructose, glucose, and sucrose were significantly lower in spears grown under warmer conditions (9.9, 7.4, 28.3 mg g^−1^ DW) compared with cooler conditions (18.9, 14.1, 37.1 mg g^−1^ DW), respectively. Although, sugar concentrations declined during storage, cooler grown spears continued to maintain significantly higher values compared with those grown under warmer conditions (Figs 3 and 4). These results agree with those reported by others, where sugar content was found to be lower in spears grown under warmer conditions possibly due to reduced substrate pools and rapid metabolism of substrates in fast growing spear tips (which have a greater propensity to fern).^4^, ^11^ During fern growth, glucose and sucrose signaling by hexokinase‐1 and trehalose‐6‐phosphate (T6P), respectively (actual concentrations not reported by Wingler^20^), are required to progress to the next stage of development such as shoot branching. However, if carbon availability is insufficient, this can lead to premature carbon depletion, which can further result in seedling death, or abortion of fruit or seed development.^20^ Sugar depletion is also reported to regulate the accumulation of an asparagine synthase (AS) synthase transcript (*pTIP12*) during spear senescence, as well as natural senescence of asparagus fern.^21^ Low carbohydrate content in asparagus tips may therefore trigger the floral buds under the bracts to abort during warmer growing conditions when growth cannot be supported by the crown or storage roots.

Sucrose concentrations at harvest have previously been suggested as a potential biomarker for predisposition to tip breakdown.^4^ In the current study, sucrose levels at harvest clearly decreased in spears grown under warmer conditions as the season progressed with values starting at ca. 90 mg g^−1^ DW (in pre‐season spears) and dropping to ca. 10 mg g^−1^ DW (in late season). A similar decrease in asparagus sucrose levels through the season has been reported elsewhere.^22^, ^23^ This coincided with steady increases in day temperature and was found to be positively correlated with an increase in sucrose synthase and acid invertase (sucrose metabolizing enzymes) activity.^22^ Soluble acid invertase activity is commonly found in rapidly expanding/growing regions and is reported to be the main enzyme responsible for sucrose breakdown in the tip regions of asparagus.^24^ The trend for glucose and fructose was not as consistent as observed in the current study and in the literature some studies reported an increase during the season^22^ whereas others showed a slight decrease.^23^ The concentration of these two sugars is, however, likely to be influenced by the rate of hexose usage rather than sucrose metabolism.^24^

Sugar content (fructose, glucose, and sucrose) was almost double in pre‐season spears (warmer grown spears with no incidence of tip breakdown) in comparison with early season spears and subsequent harvests, further supporting the hypothesis that sucrose is a potential biomarker for tip breakdown. That said, and despite the decline in fructose and glucose during storage, pre‐season spears also maintained higher values of monosaccharides at the end of storage in comparison with other harvests (approximately twofold). The lowest sucrose value of 10 mg g^−1^ DW at harvest, which was recorded in late season spears grown under warmer conditions, coincided with symptoms of tip breakdown at harvest, not previously seen in the other harvests. It is therefore possible that sucrose concentrations at harvest could give an indication of predisposition to tip breakdown, as previously proposed by Lill,^4^ although it is still unclear why.

### Asparagine unlikely to be a marker for tip breakdown

Accumulation of the amino acid asparagine, which is linked to asparagus deterioration and loss of freshness,^16^, ^24^ was found to increase significantly during storage (Fig. 4 and Table 3). There was also an overall effect of grade, where asparagine values were significantly higher in large (25.3 mg g^−1^ DW) compared to small (21.3 mg g^−1^ DW) spears. That said, and despite significant differences in tip breakdown, no differences in asparagine content were observed between the different growing conditions at harvest or during storage (Fig. 4). This is not in agreement with previous literature, which has reported asparagine accumulation in spears through the season as average temperatures increase.^23^ Moreover, total protein levels were found to be higher in spears grown under warm conditions and it was suggested that individual proteins may predispose spears to tip breakdown.^4^ Our study suggests that asparagine is unlikely to be a marker for tip breakdown, but instead it may be correlated with postharvest accumulated heat units.^25^

### Non‐structural carbohydrate content in asparagus roots is not linked to postharvest spear tip breakdown incidence

Stored carbohydrates are the main source of sugar for spear growth during the harvest period due to absence of fern,^26^ with sucrose being the primary form in which carbohydrate is translocated within the asparagus plant.^4^ Fructose, sucrose, asparagine and 1‐kestose were detected in asparagus roots (Table 4); whereas glucose levels were likely to be below the detection limit, which agrees with previous literature.^26^ At the start of the harvest period, pre‐season sugars in the roots were similar to early season samples. However, as the season progressed, fructose increased significantly (up to fourfold) in the roots (Table 4), whereas sucrose and 1‐kestose generally decreased (Table 4). This would suggest fructans were being hydrolyzed into fructose to support spear growth throughout the season, as reported by Gasecka *et al*.^26^

**Table 4:** Non‐structural carbohydrate and asparagine concentration in ‘Gijnlim’ asparagus roots

Season	Growing condition	Sucrose (mg g^−1^)	Sucrose ± SE	Fructose (mg g^−1^)	Fructose ± SE	Kestose (mg g^−1^)	Kestose ± SE	Asparagine (mg g^−1^)	Asparagine ± SE
Pre	Cooler	53.89	*2.13*	10.06	*1.44*	10.21	*0.47*	*11.51*	*0.80*
Pre	Warmer	51.82	*2.13*	12.41	*1.44*	10.75	*0.47*	*12.23*	*0.80*
Early	Cooler	53.33	*2.13*	10.47	*1.44*	9.91	*0.47*	*8.33*	*0.80*
Early	Warmer	55.53	*2.13*	16.71	*1.44*	10.88	*0.47*	*9.55*	*0.80*
Mid	Cooler	42.03	*2.46*	15.20	*1.66*	7.10	*0.54*	*12.49*	*0.92*
Mid	Warmer	39.93	*2.13*	19.31	*1.44*	6.68	*0.47*	*13.51*	*0.80*
Late	Cooler	36.50	*2.13*	23.30	*1.44*	4.63	*0.47*	*11.29*	*0.80*
Late	Warmer	43.28	*2.13*	38.76	*1.44*	9.76	*0.47*	*14.32*	*0.80*
LSD: Season		***4.6		***3.0		***1.0		***1.7	
LSD: Growing condition		*ns* (*P* > 0.05)		***2.1		***0.7		*1.2	
LSD: Season × growing condition		ns (*P* > 0.05)		***4.6		***1.5		ns (*P* > 0.05)	

Fructose, sucrose, 1‐kestose and asparagine content in asparagus roots. Concentrations (mg g⁻¹ DW) in roots sampled in 2019 at pre‐season (early April), early (late April), mid (late May), and late‐season (late June) when spears were grown under cooler and warmer conditions. Standard error of the mean (± SE) is shown in italics for each data point. Least Significant Difference (LSD) values are provided for each significant main factor and interaction (*P* < 0.05). Significance levels: ns = not significant; * *P* < 0.05; ** *P* < 0.01; *** *P* < 0.001. Total observations: 32.

Sugar and fructan content did not differ between cooler and warmer conditions, despite lower sugar levels in spears from cooler conditions and higher levels in those from warmer conditions. Generally, between the pre‐ and mid‐season harvests, the sugar profile in asparagus roots did not mirror that of the spears. However, late‐season roots grown under warmer conditions showed an increase in fructose (twofold), sucrose (1.2‐fold) and 1‐kestose (twofold) compared to cooler conditions (Table 4). Roots from asparagus crowns grown under warm conditions may have mobilized carbohydrates for spear growth, as fructans can be hydrolyzed into sucrose during growth, which is further broken down into glucose and fructose.^27^ As well as being the main sugar translocated between roots, crown, and spears, sucrose is a signal for spear growth.^26^, ^28^ These results suggest fructans were being hydrolyzed for spear growth.

Soluble carbohydrate levels in the asparagus root system are among the main factors driving growth, with observed patterns of gain and loss in the roots being used by growers to enhance yields and improve the sustainability of their crops.^29^ However, in this work the observed increase in sugars in the roots from late season spears grown in warmer conditions was not found in the spears; root sugar content therefore does not seem to reflect sugar content in the spear (which appears to be a biomarker for tip breakdown). Lower sugar concentrations in spears grown under warmer conditions may have occurred due to the plants’ inability to transport nutrients quickly during rapid growth. However, in another study, increasing crown temperatures to aid mobilizing substrates to the spear (whilst maintaining low spear temperature) did not reduce tip breakdown; instead, incidence was higher.^4^ Root sugar content is therefore unlikely to be a measure of early tip breakdown development as no differences in root sugar were observed between pre‐season versus early, and cooler versus warmer, which all had significantly different levels of tip breakdown.

## CONCLUSION

The results suggest that there is a strong association between low sugar content and higher incidence of tip breakdown; however, asparagine did not appear to be an early indicator of tip breakdown development. From the data collected, it is evident that spears became predisposed to developing tip breakdown whilst in the field. Warmer temperatures resulted in lower sugar content and increased tip breakdown. However, the two grades of spears (small vs large) had similar sugar concentrations despite having higher and lower tip breakdown incidence, respectively. Primary and secondary spear emergence from bud clusters is therefore unlikely to have an influence of predisposition to tip breakdown. The observed differences in spear sugar content between warmer temperatures and cooler temperatures were not mirrored in root sugar and fructan content. Furthermore, root sugar content was similar in the pre‐season and the early season despite pre‐season spears having almost double the concentration of sugars and no incidence of tip breakdown. This suggests that root sugar content does not provide an accurate indication of spear storage potential. In summary, although it has been demonstrated that warmer field temperatures and low sugar concentration are strong contributing factors to postharvest asparagus tip breakdown, the underlaying mechanisms responsible for tip breakdown development still need to be elucidated fully. Further research where genotypes with contrasting susceptibility to tip breakdown are grown under controlled conditions is recommended, and changes in differentially expressed genes involved in sugar metabolism could be investigated.

## AUTHOR CONTRIBUTIONS

Emma Collings: conceptualization, methodology, investigation, visualization, writing – original draft, editing. M. Carmen Alamar: Conceptualization, supervision, writing – review and editing. Leon A. Terry: conceptualization, resources, supervision, writing – review and editing.

## CONFLICT OF INTEREST

The authors declare that there are no conflicts of interest.

## DECLARATION OF GENERATIVE AI AND AI‐ASSISTED TECHNOLOGIES IN THE WRITING PROCESS

The authors declare that they have not used generative AI and AI‐assisted technology in the process of writing the manuscript.

## Supporting information


**Figure S1. Stages of tip breakdown**. A. Early stages of tip breakdown, red arrows indicate areas showing signs of darkening. B. Advanced stages of tip breakdown showing dark water‐soaked bracts.

## Data Availability

The data that support the findings of this study are openly available in CORD at https://doi.org/10.57996/cran.ceres-2697.
