# Supplementary material for: Effect of growing temperature on root carbohydrate content and postharvest asparagus tip breakdown
Source: J Sci Food Agric. 2025 Feb 6;105(8):4261–70. doi: 10.1002/jsfa.14172 (PMC12082007; doi:10.1002/jsfa.14172)
Supplement: Supplementary file 1 — Figure S1. Stages of tip breakdown. A. Early stages of tip breakdown, red arrows indicate areas showing signs of darkening. B. Advanced stages of tip breakdown showing dark water‐soaked bracts. [file JSFA-105-4261-s001.docx]

**Supplementary material for Collings et al., 2024**

**
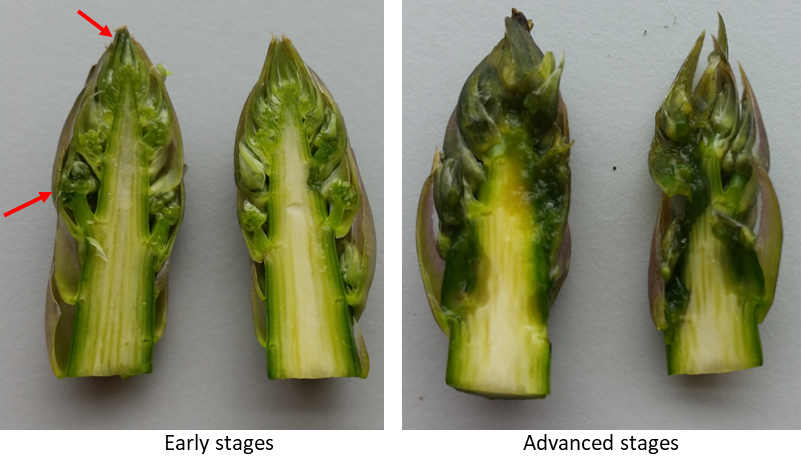
**

**B**

**A**

**Supplementary Figure 1. Stages of tip breakdown.** A. Early stages of tip breakdown, red arrows indicate areas showing signs of darkening. B. Advanced stages of tip breakdown showing dark water-soaked bracts.
